# Pelvic Abscess—A Clinical Lecture

**Published:** 1885-01

**Authors:** C. A. Lee Reed

**Affiliations:** Cincinnati; Professor of Obstetrics and Gynecology in the Cincinnati College of Medicine and Surgery


### Clinic Reports.

### PELVIC ABSCESS.

### A CLINICAL LECTURE.

By C. A. LEE REED, M. D.,
Professor of Obstetrics and Gynecology in the Cincinnati College of Medicine and Surgery.

Reported for THE ATLANTA MEDICAL AND SURGICAL JOURNAL.

There are several distinct methods of treating pelvic abscess. We have the old expectant plan—do nothing and expect your patient to get well—or die; we have treatment by evacuation, which may be accomplished by the knife, but is generally done by the aspirator, the needle being thrust either through the vaginal wall or through the abdominal wall into the abscess; and then we have the method of Tait, which is also one of evacuation, but which consists in opening the abdominal wall, stitching the abscess sac to the margins of the incision, opening the sac freely, cleansing it thoroughly and finally treating it with the drainage tube. I desire to talk about these different methods of treatment, and in doing so I shall avail myself of my private note-book for such cases as our clinic may not afford us for illustration.

The expectant plan of treatment was the one given us as our heritage by the surgery of but yesterday. It sometimes occurs to me that those grave seignors who preached that doctrine, not only in the twilight, but in the full day of modern gynecology, did so with reference to former practice, which stood in need of justification. It takes a brave man to acknowledge his errors, although they may be hidden many feet under the sod. That the let–alone policy is a mistake, particularly when adopted as an unwavering rule of practice in these cases, was and is apparent by the mortality attending its adoption. I am not prepared to give you the exact figures, for I do not know that they exist, but I do know, from a general knowledge of the literature of the subject, that the prognosis of this disease was considered much more grave under the reign of conservatism than it is to-day. That was the day when rest, hot fomentations to favor suppuration, anodynes to lull the pain while the victim's viscera were rotting, tonics to compensate for the terrible drain upon the general health, and similar measures for divers and sundry purposes were devised and applied; that was the day when it was considered that "the natural evacuation is undoubtedly the best, unless this is prócured at the expense of permanent disorganization of the pelvic viscera;" when it was thought best to wait for the abscess to *point*, and when in cases threatening to point externally, it was urged that it was "better to wait until the skin is thoroughly implicated." Do not think the day of such teaching long since past. The author who enunciated those views has but recently sent them forth again, quite unmodified, in a late American edition of his work on "Diseases of Women," while he himself commands respectful attention as teacher in one of the London schools. His doctrines, however, are of the past. They have been supplanted by the doctrines of such progressive men as the late Dr. Warren Brickell, of New Orleans, who taught that evacuation should be practiced whenever the diagnosis is made that either pus or serum is formed in the pelvis, and Tait, who first taught and practiced evacuation by free abdominal incision.

This brings me naturally to treatment by evacuation; but before I take up that subject I want to make myself a little clearer on this conservative treatment of which I have been speaking. I don't want you to think that I don't believe in rest, tonics and good diet, for they are essential concomitants of any treatment that is successful. It is well to pursue the non-intervention treatment, too—for awhile; that is, until you are sure you have a deposit in the cellular tissue. It will not be within your diagnostic ability to tell whether the accumulation is one of serum or of pus, although, by careful attention to symptomatology, you may do some creditable guessing. It is your duty to act on the supposition that it is pus and not serum, and then it is your further duty to act on the sound surgical doctrine that pus is never to be allowed in healthy tissue. But be sure of your diagnosis. I was once called to see a case under the care of another physician. He said, "Doctor, I have a case of pelvic abscess; I have watched it all along, and know it; the symptoms are urgent, and I want you to operate." I examined the case, found a fluctuating tumor to the side and back of the uterus, causing great pain, but not accompanied with arterial depression. I concluded on the instant the physician was right, and opened the sac freely, when out rushed nearly *three pints of blood.* I had plunged my bistoury into a hematocele! Five minutes' deliberation would have saved the accident, which fortunately, was not fatal. And now I think of another case, quite on the opposite extreme. A lady of 35 years consulted me at her home about pelvic troubles; from the history of her case, and from physical exploration, I was convinced she had a true pelvic abscess—quite a large one—extending well above the brim; I told her we must operate; she declined; I left. A week or ten days later a neighboring physician invited me to a *post-mortem* examination of a patient who had died the day previous from violent abdominal symptoms; it turned out to be the case to which I have just alluded. A large quantity of pus was found in the peritoneal cavity, having gotten there from the abscess sac, which had burst.

As you have doubtless already concluded, or rather as I have endeavored to conclude for you, the more valuable treatment is that which finds no tolerance for the existence of pus in the human body—permits no dalliance with nature while she leisurely impels an abscess to a "head." As conservatism* should be your law until your diagnosis is made, and made beyond a doubt, radicalism should be your watchword until you have emptied and cleansed the abscess, and cleaned it, too, to a certainty. The attainment of this result has been made easily practicable by the genius of Dieulafoy. The aspirator reduces the operation to one of minor surgical importance, but I can illustrate that fact to you better by the case here present:

Mrs. W , æt. 37, has had pronounced pelvic trouble ever since the birth of her last child, three years ago. She complains of weight and pain in the right side of the pelvis; that gives us a hint as to the location of the disease. She menstruates regularly, and without pain; that shows that the ovaries and Fallopian tubes are not involved. She complains of a throbbing, pulsating pain; that suggests to us the idea of suppuration. She said these symptoms were gradual in accession, but persistent in their development; that tells us that it is not a hematocele. Physical exploration shows the existence of a fluctuating tumor to the right and back of the uterus, pressing well down upon the vault of the vagina. Bi-manuel shows this tumor to extend slightly above the brim of the pelvis. I am

*It should be understood, in this connection, that exploratory puncture by the aspirator, or exploratory incision through the abdominal walls, for diagnostic purposes, are measures within the meaning of modern conservatism.

satisfied, from this and repeated former examinations, that this is a case of pelvic abscess.

The woman is now placed in Sims' position. The aspirator is exhausted and the needle is oiled. I want you to notice that needle; I had it made expressly for these cases; it is seven and a half inches long, and has a groove upon one side of it. The length of the instrument enables me to use it with one hand, using my index finger as a guide. I have now introduced the needle into the sac, and as I turn the stop-cock you observe that pus runs freely into the bottle. About nine ounces have been secured. A similar quantity of antiseptic water—bichloride of mercury, one to one thousand—is now placed in another bottle, into which the stopper of the aspirator with a recurrent tube is inserted. The pressure is now reversed and this water is thrown into the abscess cavity and again withdrawn. This process is repeated in this case three times, when the water comes back clear; it would have been repeated oftener if necessary to accomplish that result. What shall I now do? You would naturally say remove the needle of the aspirator; and in the present instance you would be right, but there are cases in which this would not be the final stage of the operation. You remember I called your atention to a groove upon one side of the needle which makes it practically a grooved director. Now, in some cases, I slide my bistoury up along that groove until I have made a free opening into the now emptied sac, an opening large enough to admit of the introduction of a drainage tube. Why do I not do that in this case? Because the fluid removed is, as you see, almost sero-purulent—very thin and laudable; and the anti septic fluid thrown in has, I believe, removed all that remained, and has doubtless rendered the cavity quite healthy. If, however, the pus had been thick and dark, or green, an incision would have been made and a drainage tube inserted, through which antiseptic fluids would have been thrown daily or oftener, but I'll tell you more about that after we dispose of this case. She will be kept in repose under morphia for twenty-four hours, after which she will be treated with the hot vaginal douche and still kept quiet, although, if practicable, without morphia. You noticed that I filled the vagina well with antiseptic cotton; that was done not only for its antiseptic effect, but to mechanically bring the walls of the abscess in apposition. An abdominal supporter with a pad under its lower margin on the right side is designed to supplement this mechanical device.

And now a few words more about treatment by vaginal incision.

A lady came to me a few days ago from Columbus, O., for operation upon an abscess that I had diagnosed some time previously. The aspirator was used as in this case, and an incision was made through which I introduced Thomas' dull curette and treated the cavity with some judicious roughness. I then washed it out with 1-1000 corrosive sublimate solution and introduced a drainage tube. The discharge was improved in character from the start, and gradually diminished in quantity until it disappeared on the eighth or ninth day. You may wonder why I used the curette. It was to remove from the inside of the cavity certain pathological formations that have been found to be the fountain and origin of pus in cases of recurrent abscess. The day of the pyogenic membrane is past, so it is necessary to call these structures by another name. The philologists of our profession have, I believe, slighted this subject, so, for want of a term more definite and definitive, we must speak of these formations as granulations. Byford considered it was necessary to break these down, and found upon trial that the abscesses did not recur, although he held that the remnant of the preexisting disease was a kind of cavity which filled with serum and remained inactive. I believe that by use of the curette and antisepsis, the latter of which I think Byford did not use, these cavities have been entirely obliterated in cases occurring in my own practice. Of one thing I am quite certain, and that is that without these precautions the abscesses will recur, particularly in cases of long standing in which the pus is unhealthy. This was illustrated in a case that was referred to me by my friend Dr. Skinner, of Hamilton. That case was subjected to the same treatment that you have witnessed to-day. There was some temptation to go further, but it was finally concluded to take the chances, particularly as the fluid obtained was nearly all serum ; the little pus that it contained was, however, unhealthy. This case has come back to me with a recurrence of the abscess, and I shall, in a few days, take occasion to do the job thoroughly. I mention this last case for the purpose of showing the unwisdom of not treating these conditions thoroughly at the beginning.

The radicalism of to day is the conservatism of to-morrow. This is emphatically true of gynecology. The operation I have been describing promises to be the conservatism of to-morrow, if indeed it is not of to-day. The operation of Lawson Tait, that master genius of modern gynecology, promises to take an important place in the treatment of these cases. Its range of usefulness is yet to be determined, but so far it has a brilliant record. It is my purpose to make it the special subject of a subsequent lecture, when I hope to present some interesting reports.

The question arises, when is it proper to use the aspirator, followed by the bistoury, as I have described, and when should the operation of Tait be performed? This question carries with it the presumption that each operation has its legitimate sphere, and to fully outline the field of either is not an easy task. In the operation of Tait, the greatest difficulty is experienced in those cases in which the abscesses are not large and are situated well down in the pelvic cavity. In such cases it is difficult to stitch the abscess sac to the peritoneal margins of the abdominal incisions. Now, it is in just that class of cases that the operation through the vagina is performed with the most ease and satisfaction. We might, therefore, conclude that the position and size of the accumulation has much to do with the selection of the operation. I should say that where the tumor lies along the utero-vaginal juncture and does not extend above the brim, I would select the vaginal operation; but in those cases in which I had to press hard against the vault of the vagina to reach the lower portion of the tumor, and in which the upper margin of the tumor is easily discernible above the brim, I would employ abdominal section. In the latter case there is an operation that is sometimes employed as an alternative; I mean the use of the aspirator through the abdominal wall. This is a procedure that I do not like. In the first place, the sac to be emptied is generally greatly distended, and is, in consequence, thin and friable. The act of puncture may cause, indeed has caused, rupture, with speedy fatal results. There is no opportunity of breaking down the pus-secreting granulations on the inside of the sac. There is, indeed, a general lack of facility for adopting any of these important surgical measures which we have already found so essential to success. The operation of paracentesis abdominis has received the stamp of disapproval by the leading operators in cases of ovarian cystoma; how much more emphatic should be our denunciation of the operation under conditions much more menacing to life. The usual contents of a cystic ovary are not so fatal to life when liberated into the peritoneal cavity as is the pus from one of these, or, indeed, any other abscess.

